# Retinoid receptor turnover mediated by sumoylation, ubiquitination and the valosin-containing protein is disrupted in glioblastoma

**DOI:** 10.1038/s41598-019-52696-3

**Published:** 2019-11-07

**Authors:** Virginia Rodriguez, Rolanda Bailey, Mioara Larion, Mark R. Gilbert

**Affiliations:** Neuro-oncology Branch, National Cancer Institute, National Institutes of Health, Bethesda, Maryland USA

**Keywords:** CNS cancer, Post-translational modifications

## Abstract

Resistance to therapeutic use of retinoids in glioma has been observed for over 20 years; however, the exact mechanism of resistance remains unknown. To understand retinoic acid resistance in glioma, we studied the turnover mechanism of retinoid receptor proteins in neural stem cells and glioma stem-like cells. Here, we show that in normal neural stem cells, proteasomal degradation of retinoid receptors involves sumoylation, ubiquitination and recognition by the valosin-containing protein (VCP/p97/Cdc48). We find that Sumo1 modification has a dual role to stabilize the retinoid receptor from unwanted degradation and signal additional modification via ubiquitination. Subsequently, the modified receptor binds to the VCP chaperone and both proteins are degraded by the proteasome. Additionally, we reveal that all *trans* retinoic acid (ATRA) induces VCP expression, creating a positive feedback loop that enhances degradation. In contrast, the pathway is impaired in the glioma stem-like cells resulting in the accumulation of sumoylated and high molecular weight forms of retinoid receptors that lack transcriptional activity and fail to be recognized by the proteasome. Moreover, modified receptor accumulation occurs before ATRA treatment; therefore, the transcritptional defect in glioma is due to a block in the proteasomal degradation pathway that occurs after the sumo modification step.

## Introduction

Glioblastoma (GBM) is the deadliest form of brain cancer. Despite treatment with tumor resection, radiation and chemotherapy, the median survival rate is less than 15 months^1^. Glioma stem-like cells (GSCs) are thought to be treatment resistant and drive tumor growth^2,3^. Retinoids have been successfully used to terminally differentiate the cancer stem cell population in acute promyelocytic leukemia^4^, but show only modest success in glioblastoma despite the presence of retinoid receptors in tumor cells suggesting that this cancer is resistant to retinoic acid-based therapies. An important observation in the leukemia literature is that both wild type retinoic acid receptor (RAR) and the oncogenic fusion PML-RARA protein successfully turnover in response to retinoic acid (RA)^5^. While resistance to RA can develop due to mutations in the receptor’s ligand binding domain or due to a mutation resulting in a C-terminal deletion and aberrant phosphorylation^6^, ultimately these changes would disrupt receptor protein turnover. Furthermore, without proper retinoid receptor protein turnover, the intended effect of RA to induce terminal differentiation via the classical activation of RAR, will not occur.

Retinoic acid is a metabolite of vitamin A which plays a vital role in homeostasis affecting differentiation, cell proliferation and embryogenesis. Retinoic acid binds to the retinoic acid receptor and activates the transcription of RA-specific genes. RAR forms a heterodimer with the retinoid X receptor (RXR) and binds to the retinoic acid response element (RARE) in the promoter region of various retinoic acid-induced genes^7^. RAR and RXR are encoded by three separate genes each (α, β and γ), and these receptors belong to the superfamily of nuclear receptors that includes thyroid, peroxisome proliferator-activated receptor and vitamin D receptors^8^. RARA and RXRA are the most well studied of the retinoid receptors and they can bind various structural isomers of retinoic acid. All *trans* RA binds to RAR and *9-cis* binds to both RAR and RXR receptors^9^.

Following transcription, the RA receptors are degraded by the proteasomal pathway which is necessary for optimal transcriptional activity^5,10^. The exact mechanism of the RA-receptor degradation and the role that proteasomal degradation plays in the basal protein turnover has not been elucidated. In order for the RAR and RXR to be degraded, proper posttranslational modification (PTM) must occur. Several PTMs have been observed for the RAR and RXR. For example, phosphorylation was found to be essential for the receptor’s transcriptional activity^11,12^. One of the less studied PTM is represented by sumoylation. In certain protein families, the small ubiquitin modifier (sumo) peptide plays a role in protein degradation^13^. Although sumo and ubiquitin differ in their amino acid sequences, the proteins share structural similarities, and both require a three-step enzymatic pathway to covalently attach the peptide to a lysine residue in the target protein^14^. Emerging evidence indicates that a sumo/ubiquitin hybrid signature serves as a signal for proteasomal degradation in various biological systems such as DNA repair^15^. Sumoylation of nuclear receptors is typically associated with transcriptional repression^14^, but other reports describe sumoylation as an activator of transcription^16^. Studies specific to retinoid receptors have found that the sumo modification is associated with stabilization of the receptor protein^17,18^, transport into the nucleus^19^ and may be due to inflammation^20^. However, there are no reports that sumoylation of retinoid receptors might be involved in proteasomal degradation.

Herein, we reveal that the mechanism of proteasomal degradation in retinoid receptors in normal neural stem cells involves sumoylation, ubiquitination and recognition by valosin-containing protein (VCP/p97/Cdc48). The Sumo1 modification stabilizes the receptor and signals additional modification by ubiquitination. Subsequently, the modified receptor binds to the VCP chaperone and both proteins are degraded by the proteasome. In addition, we find that all *trans* retinoic acid (ATRA) induces VCP expression producing an ATRA-VCP positive feedback loop which enhances the proteasomal degradation of the retinoid receptor. In contrast, the degradation pathway in glioma stem-like cells is impaired resulting in the accumulation of high molecular weight forms of the receptor that lack transcriptional activity and fail to be recognized by the proteasome. Moreover, the accumulation of modified retinoid receptors occurs before drug treatment; therefore, decreased retinoid receptor transcriptional activity is due to a block in the proteasomal degradation pathway that occurs after the sumo modification step. Our studies suggest that the use of combinatory therapies that target retinoid receptors and induce proteasomal degradation of the receptors to ensure protein turnover may provide a more effective therapeutic approach.

## Results

### Sumoylation of RARA occurs in normal murine neural stem cells as part of proteasomal degradation pathway, however this pathway is disrupted in glioma stem-like cells

**Figure 1:**
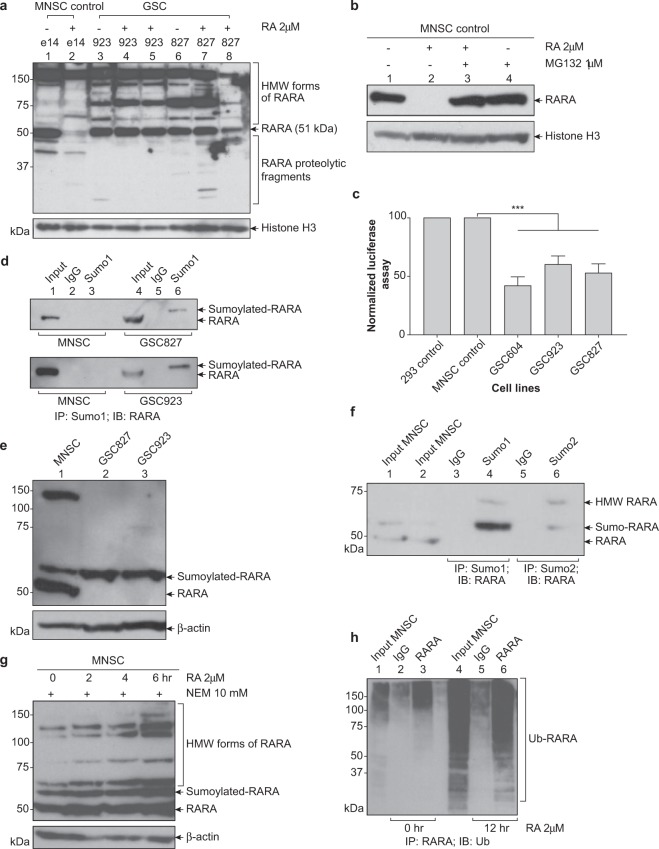
Sumo conjugation occurs in normal and glioma stem-like RARA, but glioma stem-like RARA fails to get recognized and degraded by proteasome. (**a**) RARA expression in normal and glioma stem-like cell lines. Murine neural stem cells and GSCs were treated with 2 μM all-*trans* retinoic acid for 72 h. Nuclear lysates were analyzed for RARA expression by immunoblotting. The long exposure was 10 min. (**b**) Validation that normal RARA is degraded by proteasome. MNSC were treated with 2 μM RA or 1 μM MG132, a proteasomal inhibitor, for 24 h. Nuclear lysates were analyzed for RARA expression. (**c**) RARA transcriptional activity measured by promoter luciferase assay. Several normal and GSCs were transiently transfected with a retinoic acid response element (RARE) promoter luciferase reporter for 48 h and subsequently treated with 2 μM RA for 6 h. Values for normalized luciferase activity are shown. Error bars indicate the S.E. Asterisks indicate p value < 0.001. (**d**) Endogenous GSC RARA is sumoylated by the Sumo1 peptide. Whole cell lysates were immunoprecipitated with Sumo1 antibodies or normal rabbit IgG and RARA expression was analyzed by immunoblotting. (**e**)RARA expression in presence of 10 mM NEM, a SENP inhibitor to prevent loss of sumo modification. Whole cell lysates were prepared in lysis buffer with 10 mM NEM and RARA expression was analyzed. (**f**) Endogenous RARA is sumoylated by Sumo1 and Sumo2 peptide in normal cells. MNSC whole cell lysates prepared with lysis buffer containing 10 mM NEM were immunoprecipitated with anti-Sumo1, anti-Sumo2, or normal mouse IgG and RARA expression was analyzed by immunoblotting. (**g**) Time course of RARA expression in normal cells. MNSC were treated with 2 μM RA for 0, 2, 4, 6 h and whole cell lysates were analyzed for RARA expression. (**h**) Endogenous RARA is ubiquitinated in normal cells. MNSC were treated with 2 μM RA and 1 μM MG132 for 0 and 12 h and whole cell lysates were immunoprecipitated with anti-RARA or normal rabbit IgG and ubiquitinated RARA proteins were analyzed by immunoblotting.

To determine whether retinoic acid resistance in glioma stem-like cells was due to aberrant posttranslational modification, we evaluated the protein expression levels of retinoic acid receptors. Western blot analysis of nuclear lysates showed that normal murine neural stem cells (MNSC) express the 51 kDa RARA protein and as expected, in response to treatment with all *trans* RA (ATRA), the RARA protein was down regulated (Fig. 1a). To confirm that the down regulation of RARA was due to protein degradation by the proteasomal pathway, the MNSC e14 cell line was treated with MG132, a chemical inhibitor of the proteasome. As expected, treatment with MG132 blocked the proteasomal degradation of RARA (Fig. 1b). In contrast, the addition of ATRA to human GSC923 and GSC827 did not lead to down regulation of RARA proteins, indicating that GSC 923 and GSC827 display high molecular weight (HMW) forms of RARA in presence and absence of ATRA treatment (Fig. 1a). The same pattern of RARA protein expression was observed for cytoplasmic lysates (Suppl. Fig. 1). These results show that recognition of RARA by the proteasome is lost in the GSCs and suggest that this may in part be due to aberrant posttranslational modification of RARA.

Since previous reports suggest that phosphorylation is a necessary modification for optimal transcriptional activity of RARA protein^11,12^, we determined if this modification was present in GSCs. A phospho-specific RARA antibody was not commercially available; instead 2D Western blots were used to assess the phosphorylation status of RARA in MNSC and GSC827. Western blots revealed that in response to ATRA, the GSC RARA failed to become phosphorylated (Suppl. Fig. 2) while MNSC was phosphorylated, as expected. Subsequently, we tested the transcriptional activity of several GSC RARA proteins using a promoter luciferase assay. In response to treatment with ATRA, a specific retinoic acid response element (RARE) promoter luciferase reporter demonstrated that the GSC RARA had a 40–60% decrease in transcriptional activity compared to the MNSC control (Fig. 1c). These results show that the GSC RARA lacks phosphorylation which likely contributes to its decreased transcriptional activity. In addition, the decreased RARA transcriptional function in the GSCs may be due to the accumulation of aberrant HMW forms of RARA that fail to get recognized and degraded by the proteasome.

Previous studies indicated that the HMW RARA proteins represent various sumoylated and ubiquitinated forms of RARA in other cell lines^17–19^. Immunoprecipitation and Western blot analysis showed that the endogenous RARA proteins in GSC827 and GSC923 are posttranslationally modified by the Sumo1 peptide (Fig. 1d). We hypothesized that sumoylation may have a role in regulating the proteasomal degradation of RARA during protein turnover of both normal neural stem cells and glioma stem-like cells. At first, the sumo IP did not detect the sumo modification in the normal MNSC RARA but shows sumoylated bands clearly in the GSCs. Sumoylation is a dynamic process and the covalent sumo modification can be quickly added or removed from a given target protein^21^. To determine if the lack of sumoylation in normal MNSC cells is the consequence of either inhibition or from rapid removal of sumo modification, we performed two separate experiments. In one experiment, we added N-ethylmaleimide (NEM), a chemical inhibitor of SENP proteases that prevents the removal of the sumo modification. Upon addition of NEM, the RARA Western blot now shows that the MNSCs express the 51 kDa RARA and a HMW form of the RARA protein (Fig. 1e). Endogenous Sumo immunoprecipitates (IPs) of these cells indicate that MNSC RARA is sumoylated by the Sumo1 peptide and to a lesser extent by the Sumo2 peptide (Fig. 1f). In the other experiment, we measured the time course of RA treatment in MNSC cells and observed that a sumo band appeared before additional PTM of RARA (Fig. 1g). Therefore, normal and glioma RARA are sumoylated by the Sumo1 peptide. Sumo1 modification of RARA signals additional posttranslational modification as part of the proteasomal pathway, but this pathway is disrupted in glioma.

In order to probe if sumoylation of RARA is followed by ubiquitination, we treated MNSC cells with ATRA and MG132 for 12 h. Endogenous RARA proteins were immunoprecipitated at the zero and 12 h time points followed by Western blot analyses. As expected, we observed an increase in the polyubiquitinated RARA proteins at 12 h, suggesting that sumoylation of RARA precedes additional ubiquitination and degradation via the proteasomal pathway (Fig. 1h).

### Sumo1 stabilizes RARA and regulates transcriptional activity in HEK 293 cells

**Figure 2:**
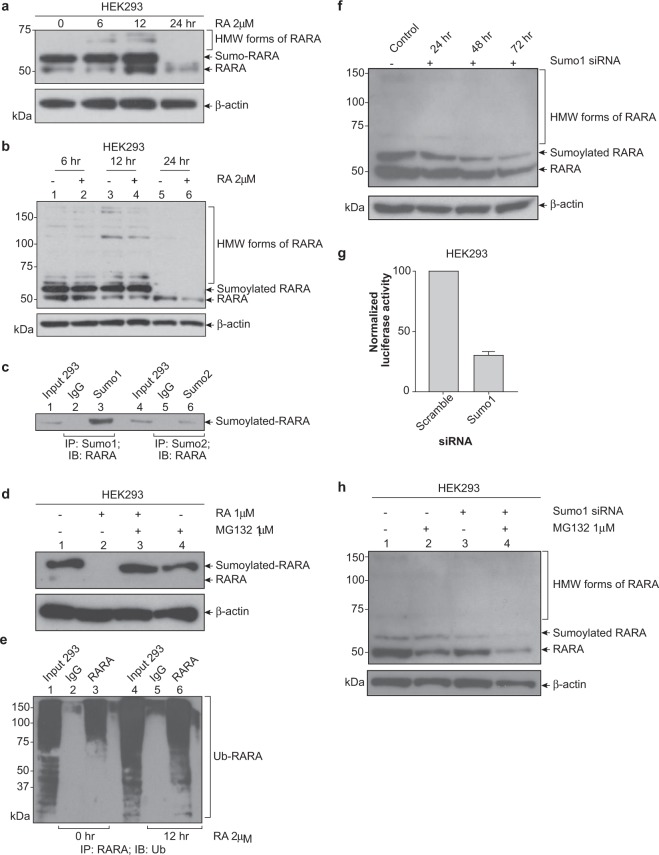
Sumo1 regulates RARA transcriptional activity and stabilizes RARA in HEK293 cells. (**a**) Time course of RARA expression in HEK293 cells. Cells were treated with 2 μM RA for 0, 6, 12, 24 h and whole cell lysates were analyzed for RARA expression. (**b**) RARA is degraded in absence of exogenous RA. HEK293 cells were treated with 2 μM RA or vehicle only for 6, 12, 24 h and whole cell lysates were analyzed for RARA expression. (**c**) Endogenous HEK293 RARA is sumoylated by Sumo1 and Sumo2 peptides. Whole cell lysates were immunoprecipitated with anti-Sumo1, anti-Sumo2 or normal rabbit IgG and RARA expression was analyzed by immunoblotting. (**d**) Normal RARA is degraded by proteasome. HEK293 cells were treated with 1 μM RA or 1 μM MG132 for 24 h. whole cell lysates were analyzed for RARA expression. (**e**) Endogenous RARA is ubiquitinated in HEK293 cells. Cells were treated with 2 μM RA and 1 μM MG132 for 0 and 12 h and whole cell lysates were immunoprecipitated with anti-RARA or normal rabbit IgG and ubiquitinated RARA proteins were analyzed by immunoblotting. The long exposure was 10 min. (**f**) Sumo1 stabilizes RARA protein. HEK293 cells treated with 0.75 μg Sumo1 siRNA for 24, 48 and 72 h. whole cell lysates were analyzed for RARA expression by immunoblotting. (**g**) Knockdown of Sumo1 results in decreased RARA transcriptional activity. HEK293 cells were co-transfected 0.75 μg Sumo1 siRNA and RARE promoter luciferase reporter for 48 h. Cells were treated with 2 μM RA for 6 h and a luciferase assay was performed. Values for normalized luciferase activity are shown. Error bars indicate the S.E. Asterisks indicate p value < 0.001. (**h**) Sumo1 protects RARA from degradation by a non-proteasomal pathway. HEK293 cells treated with 0.75 μg Sumo1 siRNA for 48 h followed by treatment with 1 μM MG132 for 12 h. Whole cell lysates were analyzed for RARA expression by immunoblotting.

To use a more robust model amenable to genetic manipulation, we evaluated the degradation of RARA in HEK293 cells. We performed a time course to study normal RARA protein expression in HEK293, the positive control cell line that expresses a transcriptionally active RARA protein as measured by the RARE promoter luciferase assay in Fig. 1c. At the zero time point, a percentage of HEK293 RARA is likely sumoylated and at the 6 and 12 h time points additional PTM occur and these modified proteins accumulate before proteasomal degradation (Fig. 2a). Interestingly, the PTM and degradation of HEK293 RARA occur in both the presence and absence of ATRA treatment (Fig. 2b) indicating that the sumo and ubiquitin modifications are likely important for basal RARA protein turnover.

Endogenous IP Western blot analyses show that HEK293 RARA is sumoylated by Sumo1 and to a lesser extent by Sumo2 (Fig. 2c). Treatment with MG132 confirmed that degradation of HEK293 RARA occurs by the proteasome (Fig. 2d). IP Western blots of HEK293 RARA treated with ATRA and MG132 show an increase in the accumulation of ubiquitinated RARA proteins at the 12 h time point (Fig. 2e). These results indicate that RARA in HEK293 cells is sumoylated and ubiquitinated, similarly to the MNSC RARA and suggest that the HEK293 represents a robust cell line that may be useful to further study the biology of RARA.

Small interfering (si)RNA was used to knockdown the expression of the Sumo1 peptide. Western blot analyses showed a decrease in the expression of the Sumo1-RARA protein as well as the 51 kDa RARA protein, suggesting that the Sumo1 peptide stabilizes the RARA protein (Fig. 2f). Co-transfection of Sumo1 siRNA and the RARE promoter luciferase reporter in HEK293 cells caused a 70% decrease in ATRA-induced transcriptional activity (Fig. 2g). Next, HEK293 cells were transfected with Sumo1 siRNA for 48 h followed by MG132 treatment for 12 h to determine if degradation of the 51 kDa RARA protein was due to the proteasome. Interestingly, loss of the Sumo1 modification does not restore the 51 kDa RARA, but causes the degradation of RARA by an alternative pathway to the proteasome (Fig. 2h). In addition, site-directed mutagenesis was used to mutate the top three sumo motifs (K399R, K171R, K161R) in the RARA protein as predicted by the SUMOplot Analysis software. Mutating the lysines to arginine maintains the correct charge but prevents the covalent attachment of the sumo peptide to the lysine residue. In response to RA, all three mutant stable cell lines had decreased transcriptional activity compared to the wild type indicating that monosumoylation can occur at each site, supporting the hypothesis that sumo modification is necessary for optimal RARA transcriptional activity (Suppl. Fig. 3).

### Proteasomal degradation of RARA involves ATRA-VCP positive feedback loop

**Figure 3:**
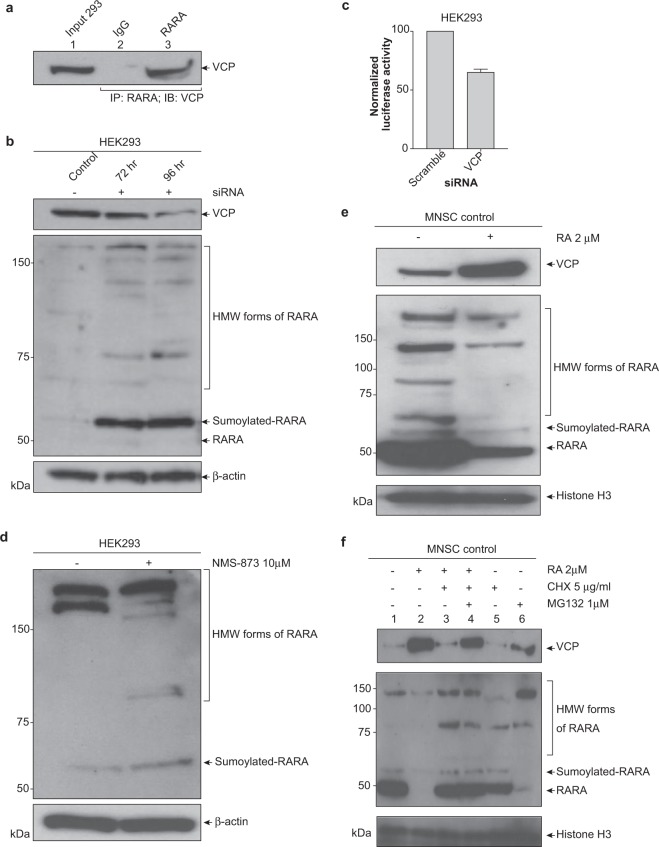
VCP binds to RARA and regulates transcriptional activity and proteasomal degradation of RARA. (**a**) Endogenous RARA binds to VCP. HEK293 whole cell lysates were immunoprecipitated with anti-RARA or normal rabbit IgG and VCP protein was analyzed by immunoblotting. (**b**) Accumulation of sumoylated and HMW forms of RARA with VCP knockdown by small interfering RNA. HEK293 cells were treated with 0.75 μg VCP siRNA for 72 and 96 h. Whole cell lysates were analyzed for RARA expression by immunoblotting. (**c**) Knockdown of VCP results in decreased RARA transcriptional activity. HEK293 were co-transfected with 0.75 μg VCP siRNA and a retinoic acid response element promoter luciferase reporter for 96 h. Cells were subsequently treated with 2 μM RA for 6 h and a luciferase assay was performed. Values for normalized luciferase activity are shown. Error bars indicate the S.E. Asterisks indicate p value < 0.001. (**d**) Increased HMW RARA with chemical inhibition of VCP. HEK293 cells were treated with 10 μM NMS-873 for 12 h and whole cell lysates were analyzed for RARA expression by immunoblotting. (**e**) ATRA increases VCP expression. MNSC were treated with 2 μM RA for 24 h and nuclear lysates were analyzed for VCP and RARA expression by immunoblotting. (**f**) ATRA-VCP feedback loop. MNSCs were treated with various combinations of 2 μM ATRA, 5 μg/ml CHX and 1 μM MG132 for 20 h and nuclear lysates were analyzed for VCP and RARA protein expression by immunoblotting.

To further understand the role of the sumo and ubiquitin signal in RARA protein turnover, we sought to identify proteins that interact with the posttranslationally modified RARA protein. In an experiment designed to identify unknown proteins that bind to all the forms of RARA, we coimmunoprecipitated proteins bound to an epitope-tagged RARA and used LC-MS/MS to identify proteins that interact with RARA protein in absence of retinoic acid treatment. The expression of the RARA-DDK protein in HEK293 was verified by Western blot (Suppl. Fig. 4). IP Western analysis showed that the DDK antibody successfully immunoprecipitated the RARA-DDK protein (Suppl. Fig. 5, top). Furthermore, as a positive control we observed that the RARA-DDK protein coimmunoprecipitated the RXRA protein, a known binding partner of RARA (Suppl. Fig. 5, bottom). The RARA-DDK IP LC-MS/MS data identified the valosin-containing protein (VCP/p97/Cdc48) as one of the proteins that interacts with RARA. To validate the LC/MS results, we immunoprecipitated endogenous RARA in HEK293 cells and probed for VCP protein by Western blot. Figure 3a (lane 3) demonstrates the presence of VCP that binds to RARA.

VCP is a member of the AAA+(ATPase associated with diverse cellular activities) ATPase family and functions in a wide range of important cellular processes involving DNA/protein interaction^22^. VCP can function as a segregase to extract a ubiquitinated or sumo/ubiquitinated target protein from a DNA/protein complex and direct it to the proteasome^23^. To determine if VCP regulates the proteasomal degradation of RARA, we used two complementary approaches. First, siRNA was employed to knockdown VCP expression in HEK293 cell. Western blot analyses showed accumulation of the sumoylated and additional HMW forms of RARA (Fig. 3b), suggesting a block in proteasomal degradation. Co-transfection of VCP siRNA and the RARE promoter luciferase reporter in HEK293 cells caused a 35% decrease in ATRA-induced transcriptional activity (Fig. 3c). Additionally, inhibition of VCP with NMS-873, a non-ATP competitive inhibitor, resulted in the accumulation of sumoylated RARA proteins (Fig. 3d). We found that treatment with ATRA alone, induced the expression of VCP (Fig. 3e).

To better understand the role of VCP in the proteasomal degradation pathway of RARA, we treated MNSCs with various combinations of ATRA, cyclohexamide (CHX) and MG132. CHX blocks protein translation which would prevent any new VCP protein expression. In response to ATRA alone, RARA decreased and VCP increased as expected. Treatment with both ATRA and CHX revealed that VCP gets degraded and this may be due to the proteasome (Fig. 3f, lane 3). The addition of MG132 which blocks the proteasome, caused VCP to accumulate (Fig. 3f, lane 4). Therefore, ATRA stimulates the production of VCP protein. Moreover, decreased VCP translation, degradation or both results in the accumulation of high molecular weight forms of the RARA that fail to be recognized by the proteasome. These results suggest that ATRA induces protein turnover of both RARA and VCP, and produces a positive feedback loop which enhances the proteasomal degradation pathway.

### Sumo1, ubiquitin and VCP also regulate the proteasomal degradation of RXRA

**Figure 4:**
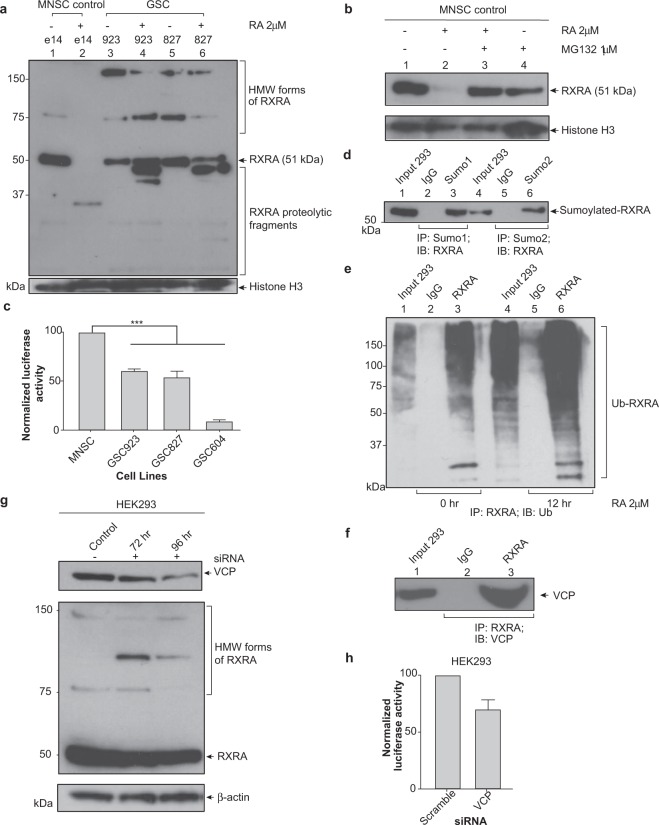
Proteasomal degradation via this pathway also applies for RXRA. (**a**) RXRA expression in normal and glioma stem-like cell lines. Murine neural stem cells and GSCs were treated with 2 μM RA for 72 h. Nuclear lysates were analyzed for RXRA expression by immunoblotting. (**b**) Validation that normal RXRA is degraded by proteasome. MNSC were treated with 1 μM RA or 1 μM MG132, a proteasomal inhibitor, for 24 h. Nuclear lysates were analyzed for RXRA expression. (**c**) RXRA transcriptional activity measured by promoter luciferase assay. Normal murine neural stem cell and GSCs were transiently transfected with a retinoic X response element promoter luciferase reporter for 48 h and subsequently treated with 2 μM *9-cis* RA for 6 h. Values for normalized luciferase activity are shown. Error bars indicate the S.E. Asterisks indicate p value < 0.001. (**d**) Endogenous HEK293 RXRA is sumoylated by Sumo1 and Sumo2 peptides. Whole cell lysates were immunoprecipitated with anti-Sumo1, anti-Sumo2 or normal rabbit IgG and RXRA expression was analyzed by immunoblotting. (**e**) Endogenous RARA is ubiquitinated in HEK293 cells. HEK293 cells were treated with 2 μM RA and 1 μM MG132 for 0 and 12 h and whole cell lysates were immunoprecipitated with anti-RXRA or normal rabbit IgG and ubiquitinated RXRA proteins were analyzed by immunoblotting. (**f**) Endogenous RXRA binds to VCP. HEK293 whole cell lysates were immunoprecipitated with anti-RXRA or normal rabbit IgG and VCP protein was analyzed by immunoblotting. (**g**) Accumulation of sumoylated and HMW forms of RXRA with VCP knockdown by small interfering RNA. HEK293 cells were treated with 0.75 μg VCP siRNA for 72 and 96 h. Whole cell lysates were analyzed for RXRA expression by immunoblotting. (**h**) Knockdown of VCP results in decreased RXRA transcriptional activity. HEK293 were co-transfected with 0.75 μg VCP siRNA and a retinoic X response element promoter luciferase reporter for 96 h. Cells were subsequently treated with 2 μM *9-cis* RA for 6 h and a luciferase assay was performed. Values for normalized luciferase activity are shown. Error bars indicate the S.E. Asterisks indicate p value < 0.001.

RARA must bind to RXRA as a heterodimer to activate RA-induced transcription^7^. RXRA belongs to the same family of nuclear receptors as RARA; however, RXRA is a master regulator that homodimerizes with itself, as well as hetrodimerizes with numerous other nuclear receptors affecting the transcriptional activation of many biological pathways^24,25^. Since the members of the nuclear receptor family have the same domain structure, we hypothesized that RXRA protein turnover would be similar to RARA, and RXRA would undergo the same PTM and degradation pathway. We analyzed the RXRA protein expression in MNSC and GSCs. The MNSC expresses the 51 kDa RXRA protein and in response to ATRA treatment, the RXRA protein is downregulated (Fig. 4a). Treatment of MNSC with MG132 confirmed that RXRA degradation is due to the proteasomal pathway (Fig. 4b). In contrast, the GSC RXRA proteins are not degraded (Fig. 4a). In response to treatment with *9-cis* RA, a specific retinoic acid response element (RXRE) promoter luciferase reporter indicates that the GSC RARA has a 40–80% decrease in transcriptional activity compared to the MNSC control (Fig. 4c). To determine if normal RXRA is sumoylated, immunoprecipitation and Western blot analyses were used to probe for sumoylated RXRA proteins. Results indicated that endogenous RXRA in HEK293 is sumoylated by both Sumo1 and Sumo2 peptides (Fig. 4d). We verified that HEK293 RXRA degradation was due to the proteasome (Suppl. Fig. 6). IP Western blot analyses of HEK293 RARA treated with ATRA and MG132 showed an increase in the accumulation of ubiquitinated RXRA proteins at the 12 h time point (Fig. 4e). Similarly, HEK293, MNSC RXRA was sumoylated by Sumo1 and Sumo2 (Suppl. Fig. 7) and ubiquitination of MNSC RXRA increased after 12 h of treatment with RA (Suppl. Fig. 8). Immunoprecipitation of endogenous RXRA in 293 binds to VCP (Fig. 4f) and knockdown of the VCP protein using siRNA causes HMW RXRA proteins to accumulate (Fig. 4g). Co-transfection of VCP siRNA and the RXRE promoter luciferase reporter in HEK293 cells showed a 30% decrease in *9-cis* RA-induced transcriptional activity (Fig. 4h). Similar to RARA, normal MNSC RXRA is sumoylated and ubiquitinated, and subsequently degraded by the proteasome. Knockdown of the VCP in HEK293 cells caused sumoylated and HMW RXRA to accumulate, suggesting that degradation is regulated by the VCP protein. Failure of RXRA protein turnover would likely affect multiple heterodimers that require RXRA and potentially alter many biological pathways.

### Working model for sumo and VCP-aided proteasomal degradation of retinoid receptors

**Figure 5:**
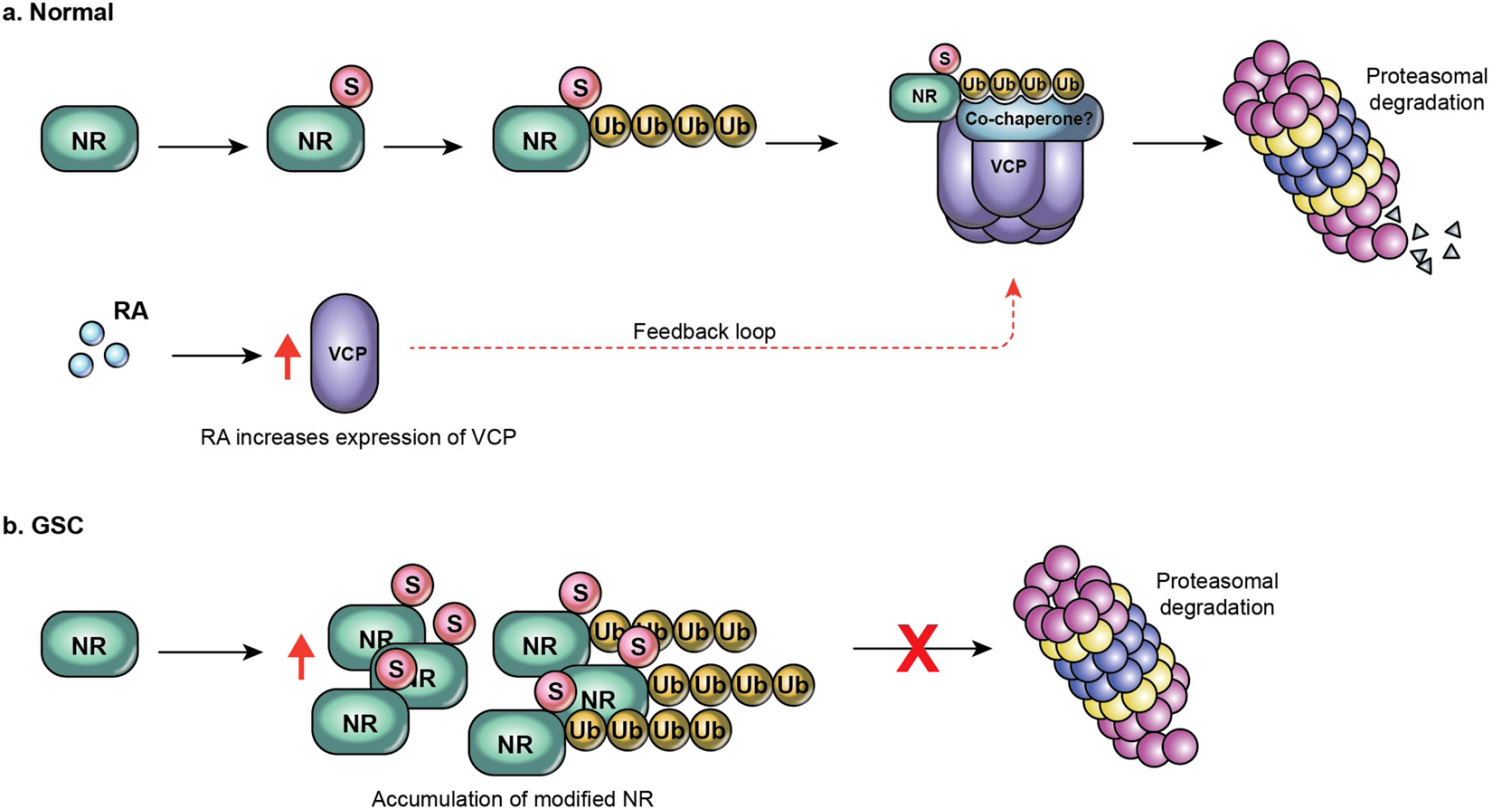
Proposed model of Sumo and VCP-assisted proteasomal degradation of posttranslationally modified nuclear receptor (NR). (**a**)In normal neural stem cells, the nuclear receptor is sumoylated by the Sumo1 peptide followed by additional posttranslational modification by ubiquitination. The VCP complex recognizes and binds to the modified receptor and delivers it to the proteasome for degradation. Additional co-chaperone protein may be involved. In addition, retinoic acid induces the expression of VCP suggesting that VCP is also degraded by the proteasome, and an ATRA-VCP feedback loop regulates the degradation of the nuclear receptor. (**b**) In glioma stem-like cells, both sumoylated and high molecular weight forms of NR accumulate due to the defect in the proteasomal degradation pathway. NR, S, Ub,VCP, RA and GSC indicate nuclear receptor, Sumo1, ubiquitin, valosin-containing protein, retinoic acid and glioma stem-like cell, respectively.

In our working model, the Sumo1 modification has a dual role: it stabilizes the RARA protein from unwanted degradation, and it serves as a signal for additional modification via ubiquitination followed by subsequent proteasomal degradation. The VCP protein assists in the degradation of the modified retinoid receptor. While VCP binds to the receptor, an unidentified VCP co-chaperone may be involved to direct VCP to the proteasome^22^. In addition, ATRA induces VCP expression creating a positive feedback loop to regulate the proteasomal degradation of RARA (Fig. 5a). In glioma stem-like cells, the retinoid receptor protein turnover process is disrupted. Although the exact defect is unknown, both RARA and RXRA fail to get recognized and degraded by the proteasome and as a consequence, accumulate in a sumoylated and HMW forms in the GSCs suggesting a common defect in the proteasomal pathway that affects these two nuclear receptors (Fig. 5b).

## Discussion

Strong evidence in the literature links catabolism of the retinoid receptor with its transcriptional activation^5,7,10^. Many studies have shown that RA induces the down regulation of the receptor and this is referred to as ligand-induced proteasomal degradation. With regards to retinoid receptors, receptor protein turnover is necessary for optimal transcriptional function. Studies have shown that site-directed mutagenesis and/or deletions at specific locations in the receptor protein prevent proper PTM, which block ligand-induced proteasomal degradation of the receptor, can disrupt DNA binding and/or interaction with the heterodimeric partner and ultimately disrupt transcriptional activity^5,7,10^. Without transcriptional activity of retinoid receptors, the intended RA-specific genes that induce terminal differentiation will not be transcribed, and therefore terminal differentiation will not take place.

RA is a successful differentiation therapy for promyelocytic leukemia^4^. Interestingly, RA induces the receptor protein turnover of both wild type RARA and the oncogenic fusion PML-RARA protein^5^. One way the patient develops resistance to RA is to acquire genetic mutations in the RARA protein that cause loss of proper PTM which results in the disruption of ligand-induced proteasomal degradation of the receptor and loss of transcriptional activity^6^. In addition, synthetic retinoids that activate RA-specific genes, but fail to down regulate the RARA or PML/RARA protein levels are unable to eradicate acute promyelocytic leukemia^26^. Perhaps the synthetic retinoids activated a retinoid receptor-independent pathway.

The exact mechanism of how the retinoid receptors are recognized and delivered to the proteasome is unknown. Here, we show that normal RARA and RXRA proteins are posttranslationally modified by sumoylation and ubiquitination, and subsequently degraded by the proteasomal pathway. Surprisingly, posttranslational modification and proteasomal degradation occurred in the absence of exogenous ligand, indicating that this process is likely necessary for basal protein turnover.

Furthermore, we found that RARA binds the valosin-containing protein (VCP/p97/Cdc48) ATPase, a segregase that extracts client proteins from a DNA/protein complex and transports them to the proteasome. Knockdown of this protein in our normal neural stem cells resulted in the accumulation of posttranslationally modified-RARA and RXRA proteins and decreased retinoic acid-induced transcription. Surprisingly, ATRA treatment alone increased VCP expression. In response to ATRA, both RARA and VCP are degraded by the proteasome, and ATRA produces a positive feedback loop between ATRA and VCP to increase the proteasomal degradation pathway.

We found that RARA and RXRA protein turnover is blocked in glioma stem-like cells and RARA transcriptional activity is disrupted. Glioma cells express sumoylated and high molecular weight RARA and RXRA proteins that have accumulated even before retinoic acid treatment. In addition, the sumoylated and HMW RARA and RXRA proteins fail to be recognized and degraded by the proteasomal pathway. Therefore, we hypothesize that the inherent resistance to retinoic acid in glioma cells may be due to a block in retinoid receptor degradation pathway that occurs after sumo modification, but before the RARA and RXRA proteins are delivered to the proteasome. Future work will focus on identifying the exact defect in the pathway which will expand our understanding of retinoic acid resistance.

Our studies provide new insight into the importance of proteasomal degradation in retinoid receptors. Contrary to the view that the RARA receptor is constitutively expressed and therefore has transcriptional activity^27^, we have demonstrated that degradation via sumo and ubiquitin is required for transcriptional activity. The pleiotropic effects of retinoic acid may have previously obscured the link between proteasomal degradation and transcriptional activity in glioma. Strong evidence indicates that RARA is degraded by the proteasomal pathway^5^, and protein turnover is linked to the receptor’s transcriptional activity^10^. Perhaps disruption to the proteasomal processing of RARA was missed in glioma because the receptor-independent effects of RA were unknown at that time^28,29^. While the glioma cells appeared to differentiate in response to retinoid treatment^27^, a lack of degradation of RARA in response to RA treatment would indicate a defect in protein turnover and RARA transcriptional function rather than constitutive activity.

To understand retinoic acid resistance in glioma, we studied the retinoid receptors in normal neural stem cells and glioma stem-like cells. We found that the turnover mechanism of retinoid receptors is disrupted in glioma. The glioma stem-like cells express high molecular weight forms of RARA and RXRA that fail to be recognized by the proteasome and lack transcriptional activity. Modified retinoid receptors accumulate before drug treatment, indicating that the cancer cells likely have a general defect in the proteasomal degradation pathway that occurs after sumo modification. Since RXRA dimerizes with other nuclear receptors, disruption of RXRA protein turnover would potentially affect multiple biological pathways. The defect in protein processing that leads to the accumulation of sumoylated retinoid receptors may similarly impact many other short-lived proteins and may help explain the presence of increased global sumoylation in glioma cells reported previously^30^. We found that Sumo1 and VCP regulate the transcriptional activity and proteasomal degradation of the retinoid receptors RARA and RXRA in normal cells. The Sumo1 modification stabilizes the receptor from unwanted degradation, and serves as a signal for additional modification by ubiquitination which is then recognized by the VCP complex for proteasomal degradation. The ATRA-VCP positive feedback loop provides another layer of regulation in RARA protein turnover. These findings highlight the importance of posttranslational modifications involved with proteasomal degradation of the retinoid receptors, enabling a better understanding and potential therapeutic targeting of this important family of receptors.

## Methods

### Cell lines and reagents

HEK293 and HEK293T cell lines were purchased from American Type Culture Collection. Several glioma stem-like cell lines derived from both primary (GSC923) and recurrent (GSC827, GSC604) glioblastoma tumors and normal murine neural stem cells (MNSC) were described previously^31^. All cells were grown at 37 °C in 5% CO_2_. HEK293 cells were cultured in DMEM supplemented with 10% FBS, 2 mM L-glutamine and 1% penicillin-streptomycin (Gibco), and the GSC and MNSC were cultured in NBE complete media^31^. Chemical such as MG132, N-ethylmaleimide, NMS-873, cyclohexamide and ATRA were purchased from Sigma-Aldrich. Puromycin was from Life Sciences. Antibodies used in Western blots and immunoprecipitations included: RARA, RXRA, Sumo1, Sumo2, Ub, VCP from Santa Cruz Biotechnology, DDK from Origene, β-actin from Sigma-Aldrich, normal rabbit IgG and normal mouse IgG from Dako, and Histone H3 from Cell Signaling.

### Western blot and immunoprecipitation

Cells were collected by scraping plates in PBS and centrifuging 1500 rpm, 3 min. Cell pellets were lysed with cold 1X Cell Signaling lysis buffer from Cell Signaling with fresh protease inhibitors and PMSF from Sigma-Aldrich. When indicated, 10 mM NEM was added to lysis buffer. Cells were incubated on ice for at least 10 min and centrifuged 13,200 rpm, 10 min at 4 °C. Supernatants were transferred to a fresh tube and protein concentrations were quantified using the Pierce BCA kit from ThermoFisher. Typically, 30 μg of whole cell lysate was loaded on a 4–12% Bis-Tris NuPAGE gel (Invitrogen) for Western blots. Before loading on the gel, an aliquot of 4X LDS loading buffer and 10X DTT (Invitrogen) was added for a final concentration of 1X each, sample was heated 5 min, 99 °C. Subcellular fractionations were prepared using the ProteoExtract subcellular extraction kit purchased from Calbiochem.

For immunoprecipitations of endogenous protein, 2 mg of whole cell lysate prepared in 1X Cell Signaling lysis buffer with fresh protease inhibitors and PMSF was incubated with 20 μl of a slurry of antibody covalently bound to agarose beads. Solution was incubated overnight at 4 °C with end-over-end shaking. Complexes were centrifuged 3000 rpm, 1 min at 4 °C and washed 3X with 1X lysis buffer. Pellet was resuspended in 30 μl of 1X LDS loading buffer that contained 1X DTT and heated 5 min, 99 °C centrifuged 3000 rpm, 5 min at room temperature, and supernatant loaded on gel.

After loading samples on gel, electrophoresis was performed using MOPS buffer (Invitrogen) and proteins were transferred to PVDF membrane (Invitrogen), blocked for 1 h in 5% blocking solution from BioRad in 1X TBST (Teknova) from and then primary antibody (1:1000) was added and incubated overnight at 4 °C with gentle shaking. Blots were washed 3 × 5 min each in 1X TBST and incubated with appropriate secondary antibody for 1 h at room temperature. Blots were washed 3 × 5 min each in 1X TBST and SuperSignal West chemiluminescence (ThermoFisher) was used to develop signal and blots were exposed to Amersham Hyperfilm ECL (GE).

For immunoprecipitations of DDK-tagged RARA protein, cells were lysed in IP buffer (Cell Signaling) and 200 μg of whole cell lysate was mixed with 20 μl of anti-DDK magnetic bead slurry (Origene) in 1X lysis buffer and incubated for 2 h at 4 °C with end-over end shaking. Complexes were pelleted using a magnetic stand and washed 3 times with 1X lysis buffer. Pellet was resuspended in 30 μl 1X LDS loading buffer with 1X DTT, heated for 5 min at 99 °C, beads were pelleted with magnet, and supernatant was loaded on gel for Western blot or liquid chromatography– mass spectrometry (LC-MS) analysis.

### 2D Western blot

Cells were lysed in IEF lysis buffer (8 M urea, 1% NP-40, 10 mM DTT) with fresh protease and phosphatase inhibitors (Sigma-Aldrich). Proteins were separated by isoelectric focusing using the Ettan IPGphor II isoelectric focusing system (GE) and separated by molecular weight using 4–12% Bis-Tris ZOOM NuPAGE gel (Invitrogen). A 7 cm Immobiline DryStrip (pH 3–10) was rehydrated in 0.5% IPG DeStreak rehydration buffer for 24 h (GE). Two hundred micrograms of whole cell lysate were mixed with DeStreak buffer and loaded on a Immobiline DryStrip, and IEF was performed according to manufacturer’s instructions. Following IEF, the strip was equilibrated in 1X LDS loading buffer with 1X DTT (Invitrogen) and loaded into the single well of the ZOOM NuPAGE gel and the standard Western protocol was followed.

### RNA interference

For siRNA transfections, cells were seeded into six well dishes at a density of 200,000 cells/well. The following day, cells were transfected with 0.75 μg of siRNA or scramble negative control using SCBT transfection solution and transfection media as per manufacturer’s instructions. Whole cell lysates at 48, 72, and 96 h were prepared and analyzed by Western blot.

For lentiviral clones, the RARA-DDK construct and empty vector negative control were purchased from Origene. HEK293T cells were transfected with 1 μg of plasmid and 1 μg of lentiviral packaging components using Lipofectamine 2000 protocol. After 48 h, supernatants were collected, stored at 4 °C and fresh media was added to plates. Another round of supernatants was collected, pooled with the first and virus was concentrated using Lenti-X concentrator (Clontech). Lenti-X GoStix were used to determine a MOI of 5 × 10^5^ IFU/ml (Clontech). HEK293 and GSC923 cells were transduced with 20 μl of concentrated virus and stable clones were selected with puromycin 2 μg/μl.

### Luciferase assay

HEK293, MNSC and GSCs were seeded at a density of 100,000/well in 12 well dishes. The following day, cells were transfected with 1.5 μg of RARE plasmid (Qiagen) or controls using the standard Lipofectamine 2000 protocol (Invitrogen). After 48 h, cells were treated 2 μM ATRA for 6 h at 37 °C. Following the manufacture’s protocol, cells were harvested and processed using the Promega luciferase reagent kit (Promega). The QuikChange site-directed mutagenesis kit (Thermo Fisher) was used to generate RARA sumo motif mutants and the mutations were verified by sequencing.

### LC-MS/MS

Immunoprecipitated sample was run on a 4–12% Bis–Tris NuPAGE gel (Novex, Life Technologies). The proteins on the SDS-PAGE were stained with SimpleBlue Safestain (Invitrogen). Gel bands were exercised continuously for the entire gel lane. The gel bands stained with Coommassie Blue were performed with in-gel tryptic digestion to extract the peptides^32^. In brief, each gel band was destained with 25 mM NH_4_HCO_3_, 50% acetonitrile, pH 8.3. After lyophilization, the dried gel bands were incubated with 100 μL of trypsin solution (20 μg/mL, Promega) at 37 °C for 16 hours. The peptides in the gel bands were extracted with 70% acetonitrile, 5% formic acid. Each peptide sample was lyophilized and desalted by C18 ZipTip (Millipore) for liquid chromatography– mass spectrometry (LC-MS) analysis.

### MS analysis and data processing

Each sample (6 μL) was loaded on an Easy nLC II nano-capillary HPLC system (Thermo Scientific) with a C18 Nano Trap Column, (Thermo Scientific) and an C18 Nano analytical column (15 cm, nanoViper, Thermo Scientific) connected with a stainless steel emitter, coupled online with a Q Exactive hybrid OrbiTrap mass spectrometer (Thermo Scientific) for MS analysis. Peptides were eluted using a linear gradient of 2% mobile phase B (acetonitrile with 0.1% formic acid) to 42% mobile phase B within 45 min at a constant flow rate of 200 nL/min. The ten most intense molecular ions in the MS scan were sequentially selected for high-energy collisional dissociation (HCD) using a normalized collision energy of 30%. The mass spectra were acquired at the mass range of m/z 350–2000. Nanospray Flex™ Ion Sources (Thermo Scientific) capillary voltage and temperature were set at 1.7 kV and 300 °C, respectively. The dynamic exclusion function on the mass spectrometer was enabled during the MS2 data acquisition. The MS data were searched against Homo sapiens protein database downloaded from the European Bioinformatics Institute website utilizing SEQUEST HT interfaced with Proteome Discoverer 1.4 (Thermo Scientific). Up to two missed tryptic cleavage sites was allowed and oxidation (+15.9949 Da) of methionyl residue was included as a dynamic modification. The precursor ion tolerance was set at 20 ppm and the fragment ion tolerance was set at 0.05 Da. The peptide identifications are filtered through protein percolator with the cutoff of a false peptide discover rate (FDR) less than 1% for all peptides identified. Each protein identified was compared semi-quantitatively under different treatment conditions with the total peptide count or total precursor ion peak area integration.

### Differentiation assay

HEK 293 were seeded at a density of 3 × 10^6^ cells/plate in 10 cm dishes. MNSC and GSC neurospheres were trypsinized to obtain a single cell suspension and seeded at the same cell density on polyornithine (Sigma-Aldrich) coated 15 cm dishes. The following day, media was changed to DMEM supplemented with 5% FBS and 2 μM ATRA. Cells were incubated at 37 °C at designated times. If cells were incubated for longer than 48 h, the media was removed and replaced with fresh media.

### Statistical analysis

All experiments were repeated at least three times. An unpaired student’s T-test was used to determine the P-value for the RARE and RXRE promoter luciferase assays.

## Supplementary information

Sumo1 and valosin-containing protein (VCP/p97/Cdc48) regulate retinoid receptor protein turnover– a process disrupted in glioblastoma

## Data Availability

The datasets generated and/or analyzed during the current study are available from the corresponding author on reasonable request.
