# Supplementary material for: Retinoid receptor turnover mediated by sumoylation, ubiquitination and the valosin-containing protein is disrupted in glioblastoma
Source: Sci Rep. 2019 Nov 7;9:16250. doi: 10.1038/s41598-019-52696-3 (PMC6838077; doi:10.1038/s41598-019-52696-3)

# Retinoid receptor turnover mediated by sumoylation, ubiquitination and the valosin-containing protein is disrupted in glioblastoma

Virginia Rodriguez, Rolanda Bailey, Mioara Larion, Mark R. Gilbert

Supplemental Information

**Supplemental Figure 1**. **In response to RA, glioma stem-like RARA protein fails to get recognized and degraded by proteasome.** RARA expression in normal and glioma stem-like cell lines. Murine neural stem cells and GSCs were treated with 2 μM all-*trans* retinoic acid for 72 h. Cytoplasmic lysates were analyzed for RARA expression by immunoblotting. The long exposure was 10 min.

**Supplemental Figure 2. GSC827 RARA lacks phosphorylation.** Murine neural stem cells and GSC827 were treated with 2 μM all-*trans* retinoic acid for 48 h. Whole cell lysates were analyzed for RARA expression by immunoblotting using two-dimensional gel electrophoresis.

**Supplemental Figure 3. RARA sumo motif mutants have decreased RARA transcriptional activity.** HEK293 stable cell lines expressing wild type RARA or three separate sumo motif mutants (K399R, K171R, K161R) were transiently transfected with a retinoic acid response element promoter luciferase reporter for 48 h and treated with 2 μM RA for 6 h. Values for normalized luciferase activity are shown. Error bars indicate the S.E. Asterisks indicate p value < 0.001.

**Supplemental Figure 4. HEK293_RARA_DDK stable cell line expresses DDK-tagged RARA protein.** Whole cell lysates were analyzed for DDK expression by immunoblotting.

**Supplemental Figure 5. HEK293 DDK-tagged RARA protein immunoprecipitated by DDK magnetic beads (top).** HEK293 cells were lysed in IP buffer and 200 μg of whole cell lysate was immunoprecipitated with anti-DDK magnetic beads, and samples were analyzed for DDK expression. **(bottom) HEK293 DDK-tagged RARA binds to heterodimeric partner, RXRA protein.** HEK293 cells were lysed in IP buffer and 200 μg of whole cell lysate was immunoprecipitated with 20 μl of anti-DDK magnetic bead slurry, and samples were analyzed for RXRA expression.

**Supplemental Figure 6. HEK293 RXRA is sensitive to proteasomal degradation.** HEK293 cells were treated with 1 μM RA or 1 μM MG132, a proteasomal inhibitor, for 24 h. Nuclear lysates were analyzed for RXRA expression.

**Supplemental Figure 7. Murine neural stem cell RXRA is sumoylated by Sumo1 and Sumo2 peptides.** Endogenous RXRA is sumoylated by Sumo1 and Sumo2 peptide in normal cells. MNSC whole cell lysates were immunoprecipitated with anti-Sumo1, anti-Sumo2 or normal rabbit IgG and RXRA expression was analyzed by immunoblotting.

**Supplemental Figure 8. Expression of Ubiquitinated-RXRA proteins increase from zero to 12 h after treatment with RA.** Endogenous RXRA is ubiquitinated in normal cells. MNSC were treated with 2 μM RA and 1 μM MG132 for 0 and 12 h and whole cell lysates were

immunoprecipitated with anti-RXRA or normal rabbit IgG and ubiquitinated RXRA proteins were analyzed by immunoblotting. The long exposure was 5 min.

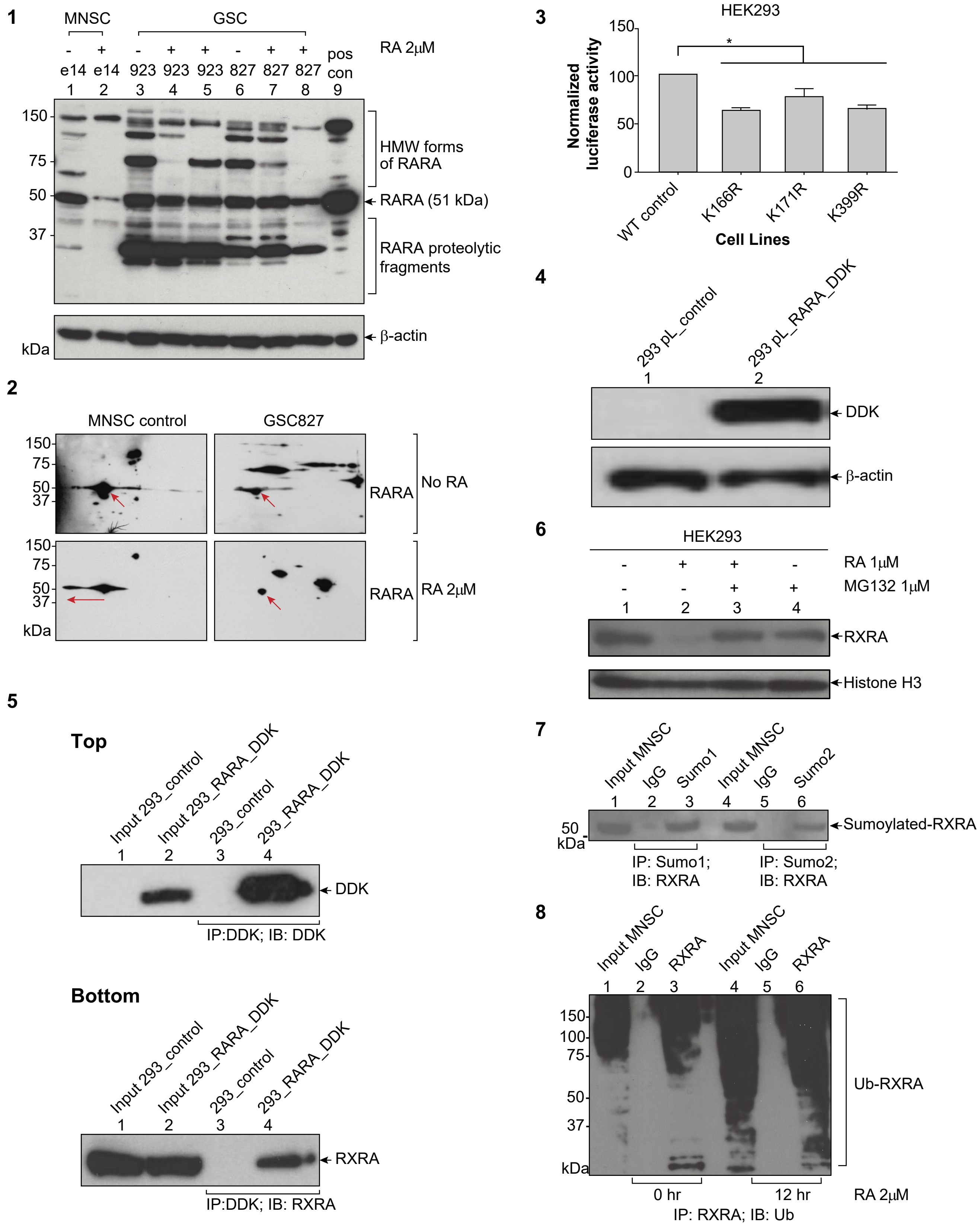
1
MNSC
GSC
- + - + + - + +
RA 2μM
e14 e14 923 923 923 827 827 827 pos con
1 2 3 4 5 6 7 8 9
150
75
50
37
kDa
HMW forms of RARA
RARA (51 kDa)
RARA proteolytic fragments
β-actin
2
MNSC control
GSC827
150
75
50
37
kDa
RARA
No RA
RARA
RA 2μM
3
HEK293
Normalized luciferase activity
150
100
50
0
*
WT control
K166R
K171R
K399R
Cell Lines
4
293 pL_control
293 pL_RARA_DDK
1
2
DDK
β-actin
5
Top
Input 293_control
Input 293_RARA_DDK
293_control
293_RARA_DDK
1 2 3 4
DDK
IP:DDK; IB: DDK
Bottom
Input 293_control
Input 293_RARA_DDK
293_control
293_RARA_DDK
1 2 3 4
RXRA
IP:DDK; IB: RXRA
6
HEK293
- + + -
RA 1μM
- - + +
MG132 1μM
1 2 3 4
RXRA
Histone H3
7
Input MNSC
IgG
Sumo1
Input MNSC
IgG
Sumo2
1 2 3 4 5 6
50
kDa
Sumoylated-RXRA
IP: Sumo1; IB: RXRA
IP: Sumo2; IB: RXRA
8
Input MNSC
IgG
RXRA
Input MNSC
IgG
RXRA
1 2 3 4 5 6
150
100
75
50
37
kDa
Ub-RXRA
0 hr
12 hr
RA 2μM
IP: RXRA; IB: Ub

Figure 1a

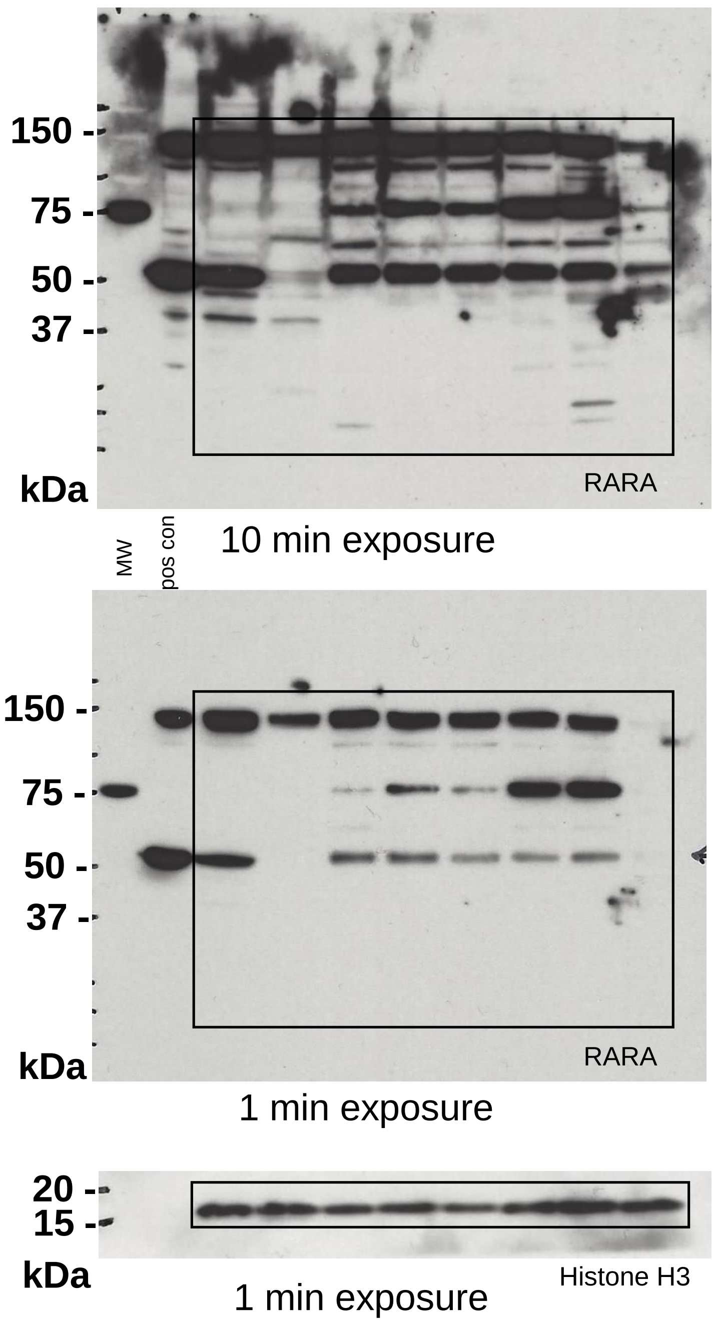


Figure 1b

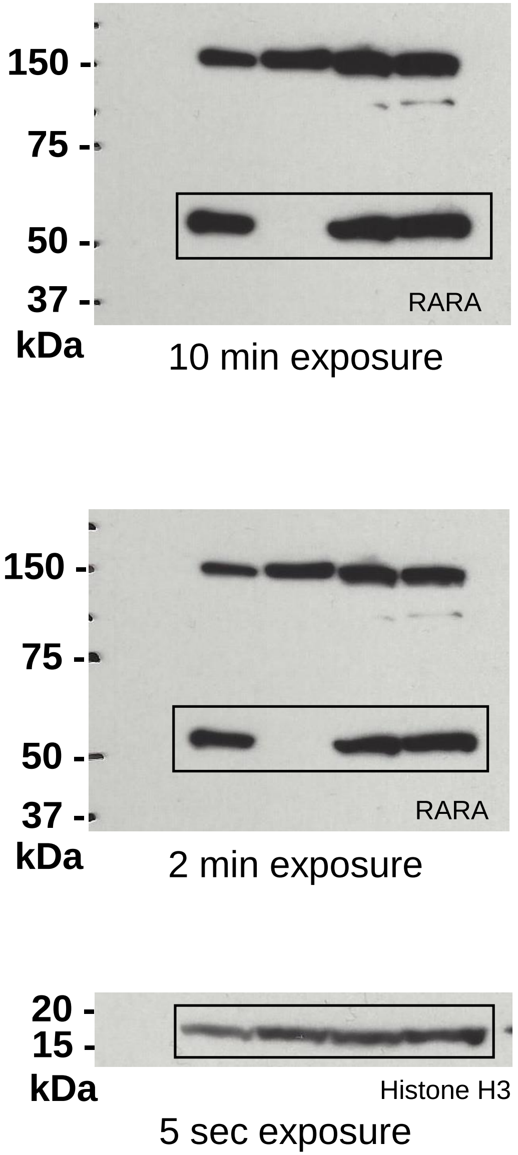


Figure 1d upper panel

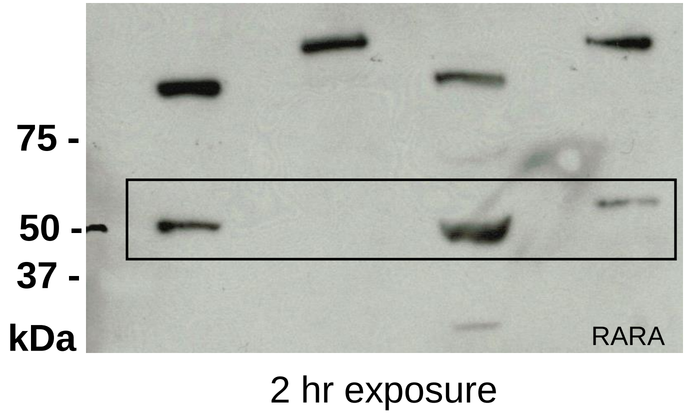


Figure 1d lower panel

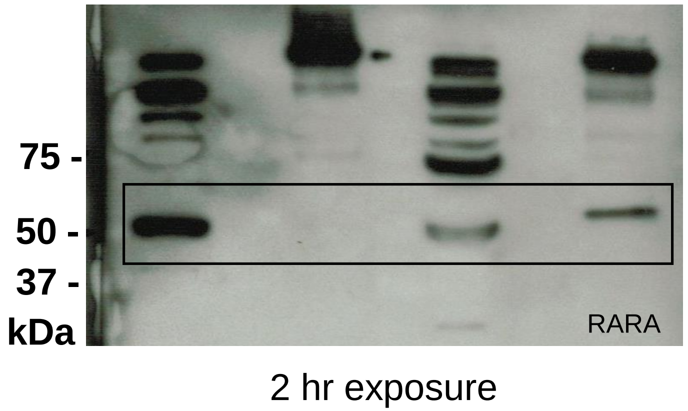

## Figure 1e

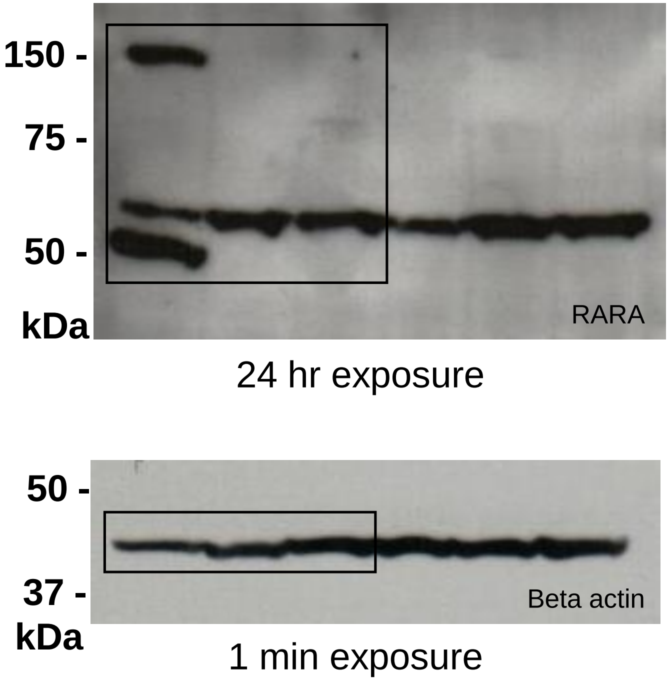


## Figure 1f

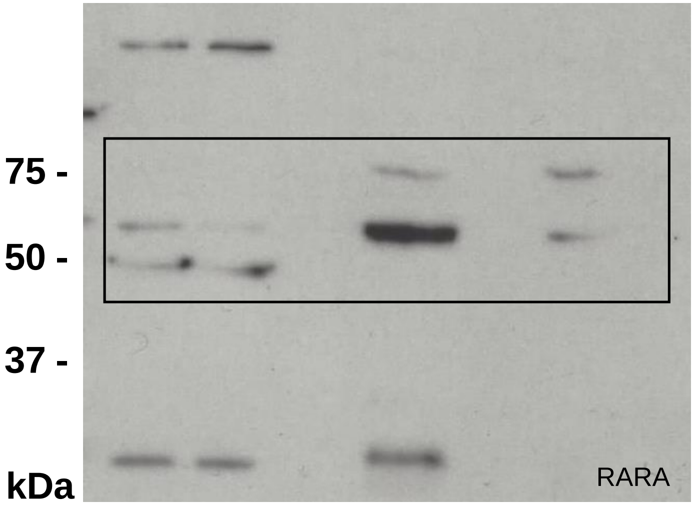


24 hr exposure

## Figure 1h

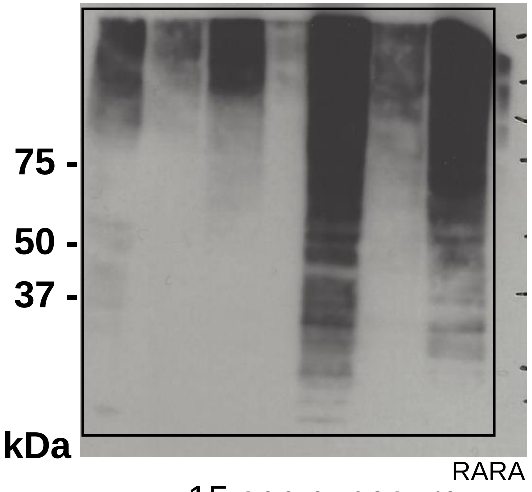


15 sec exposure

## Figure 1g

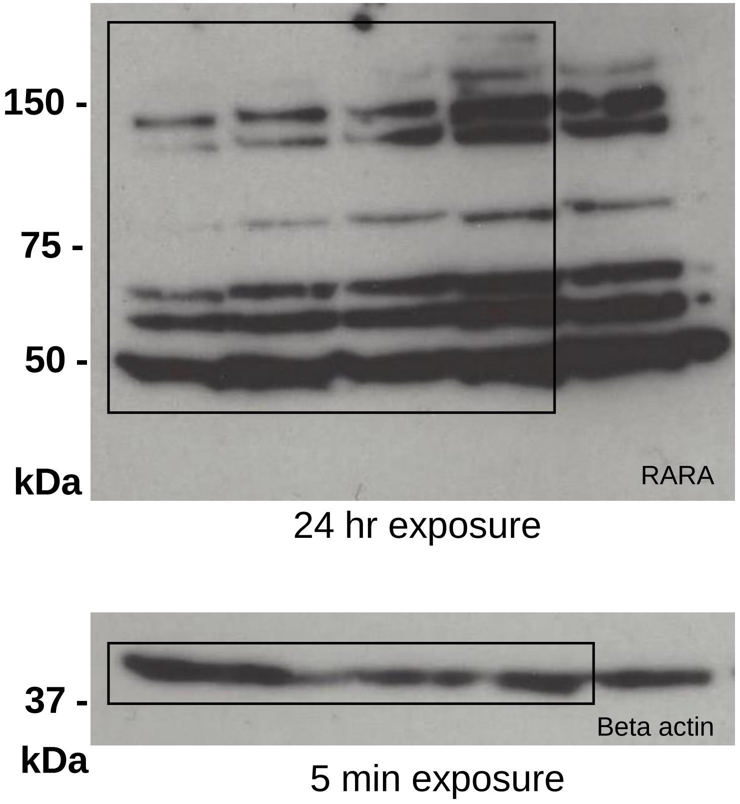

Figure 2a

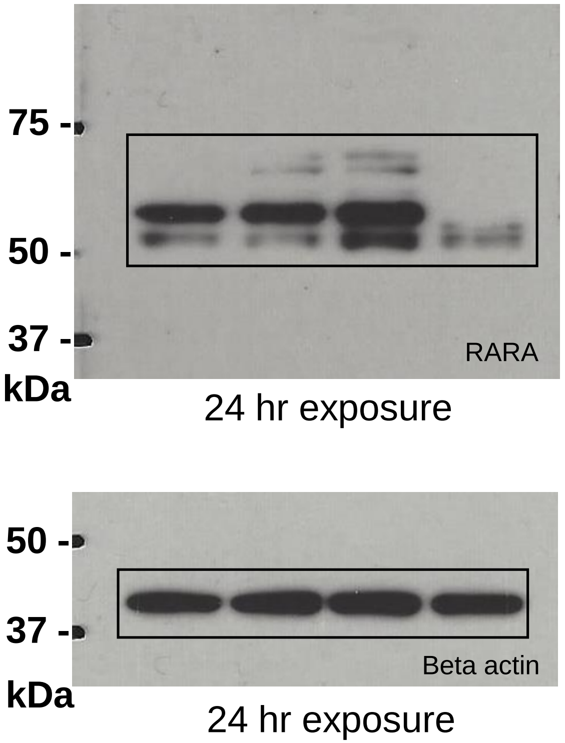


Figure 2b

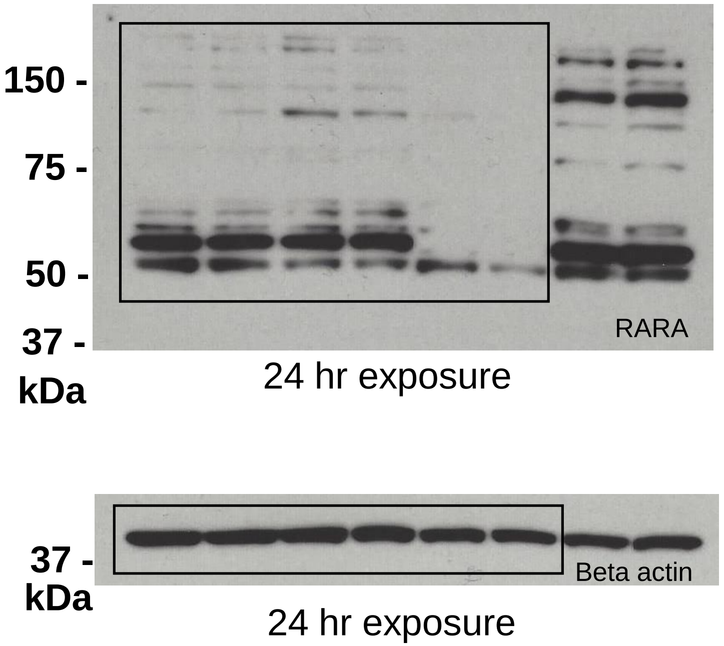


Figure 2c

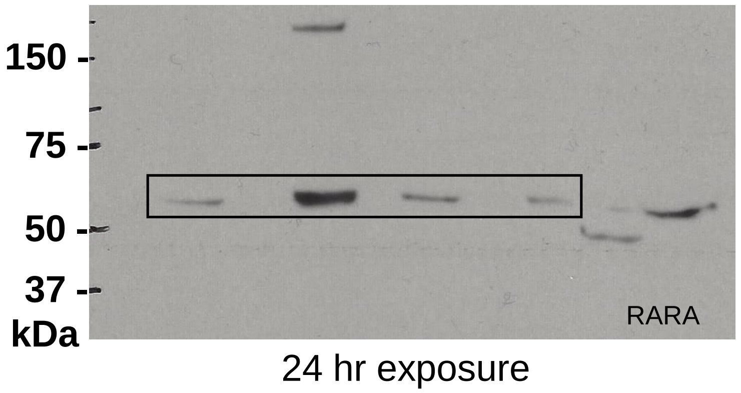


Figure 2d

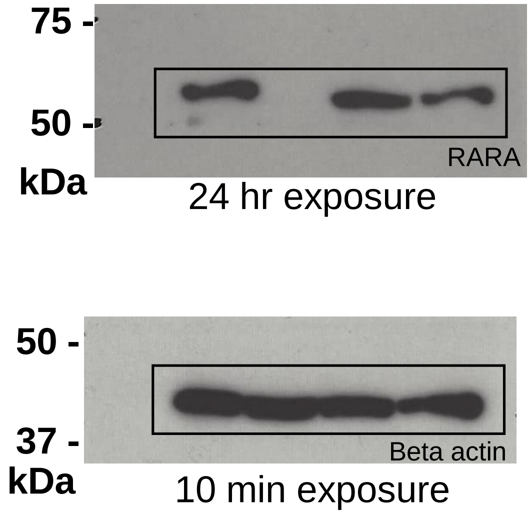

## Figure 2e

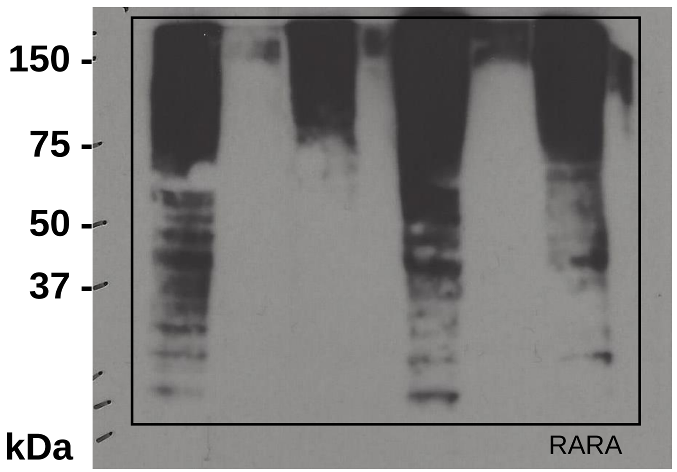


3 min exposure

## Figure 2f

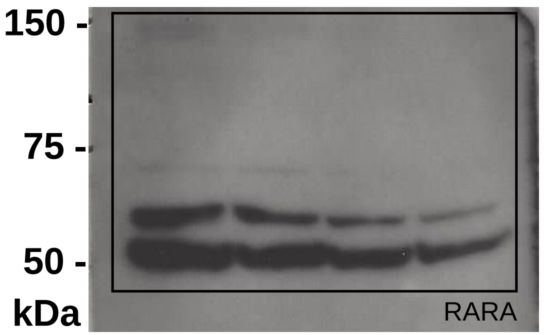


24 hr exposure

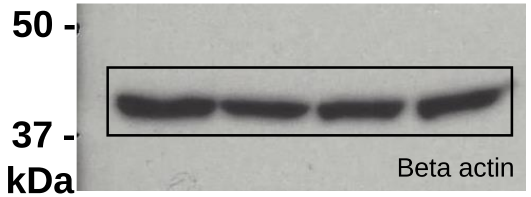


5 min exposure

## Figure 2h

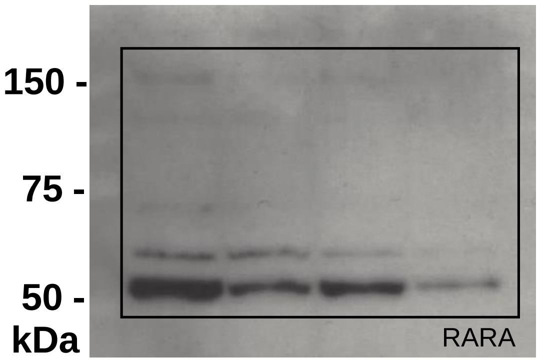


24 hr exposure

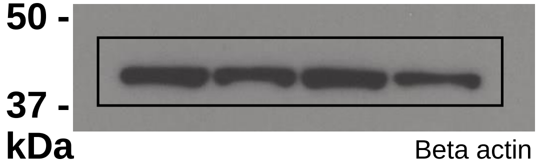


5 min exposure

## Figure 3a

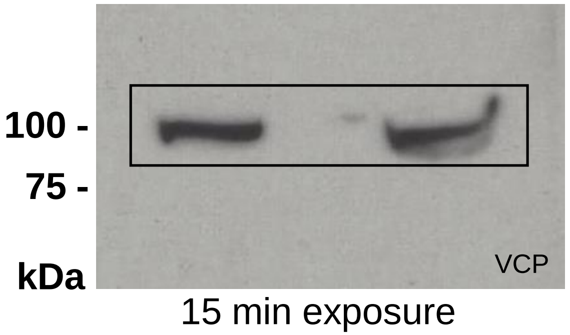


## Figure 3b

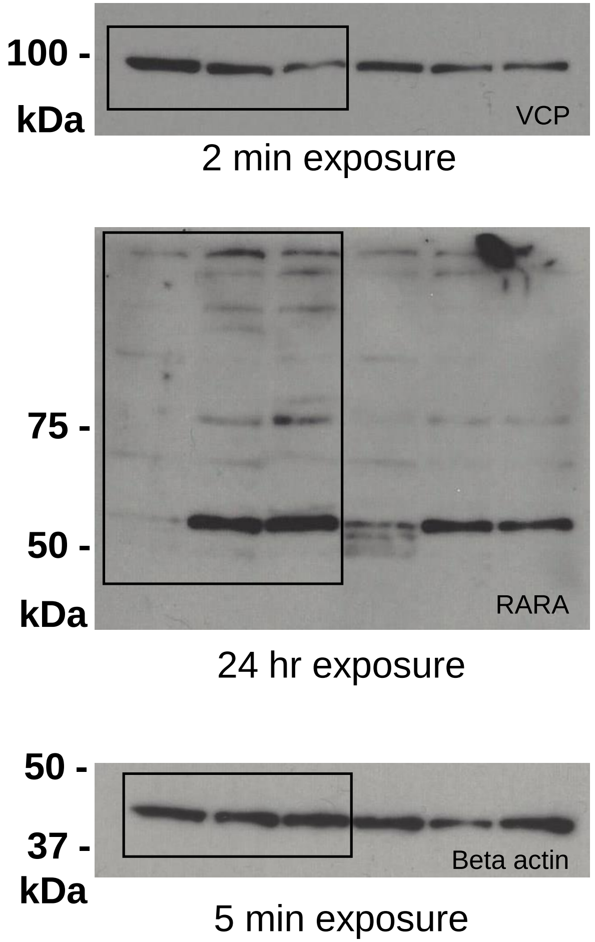


## Figure 3d

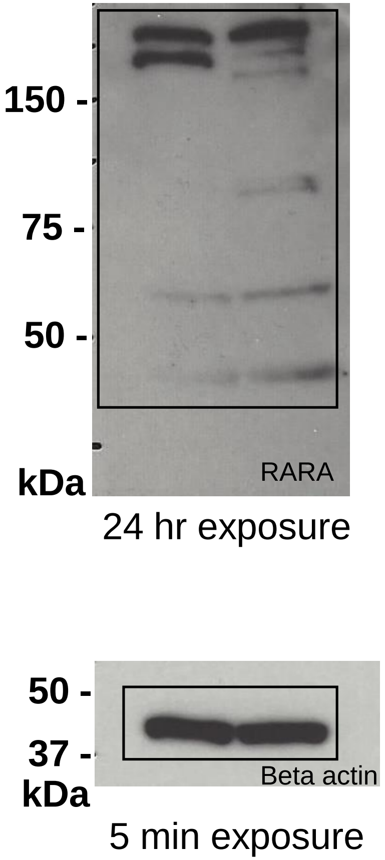


## Figure 3e

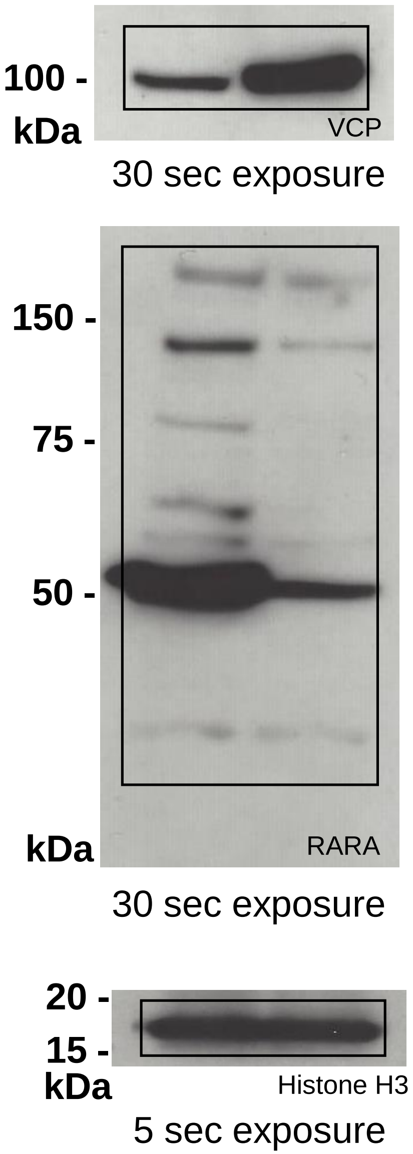


## Figure 3f

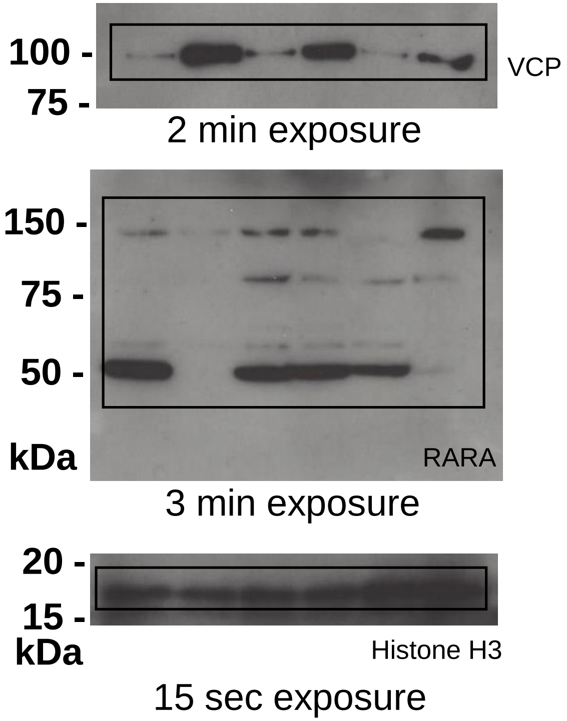

## Figure 4a

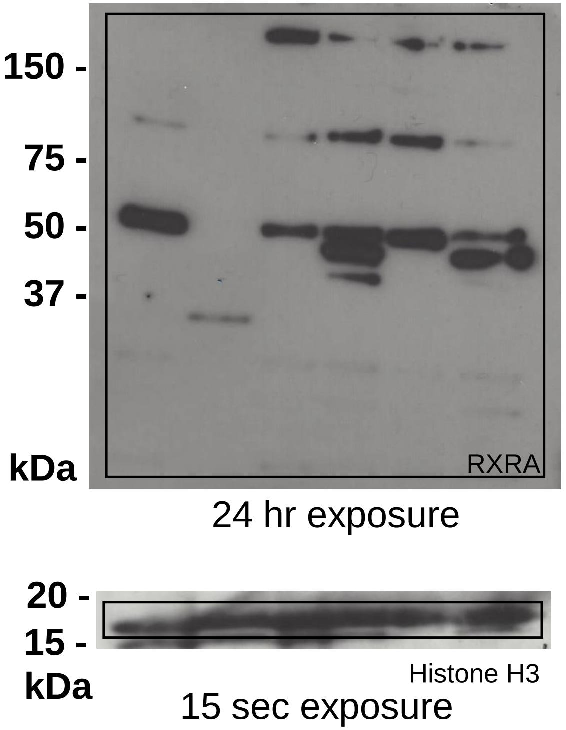


## Figure 4b

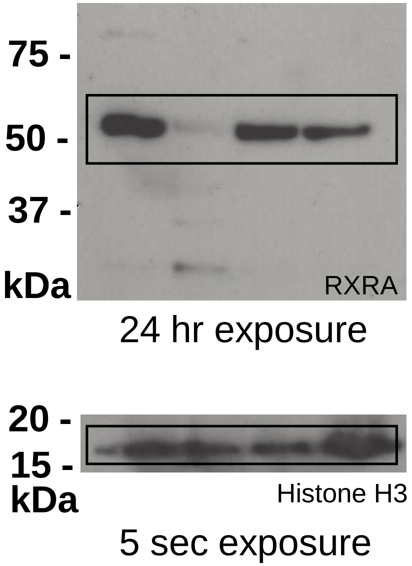


## Figure 4d

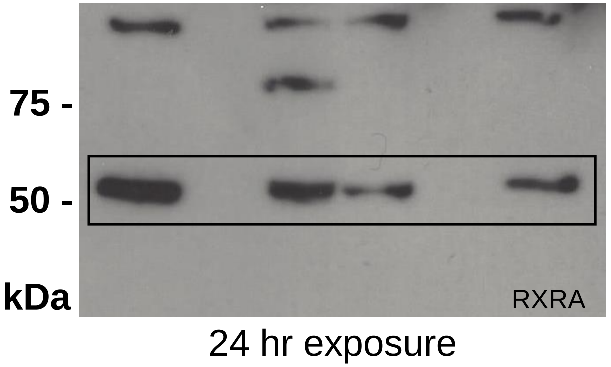


## Figure 4e

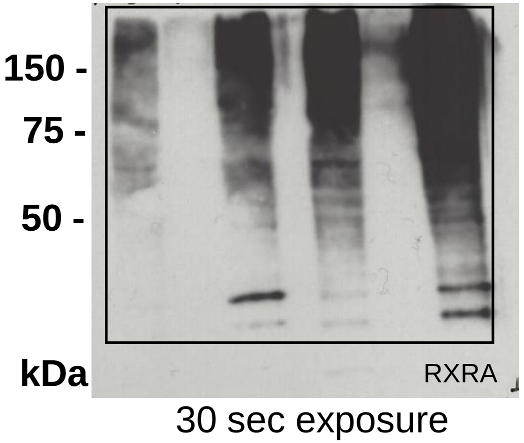


## Figure 4f

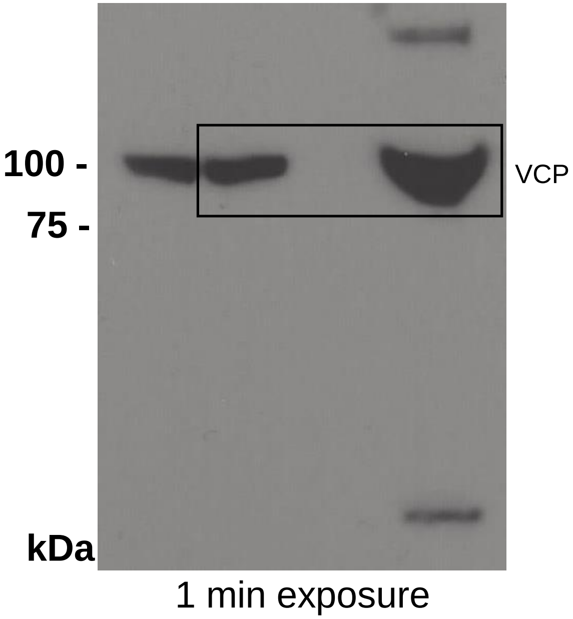


## Figure 4g

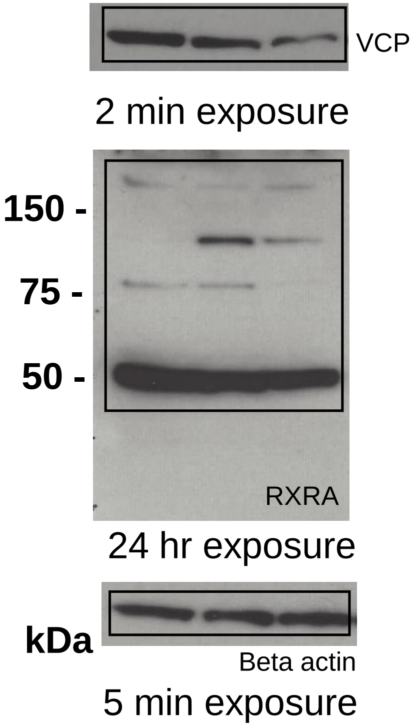

## Supplemental Figure 1

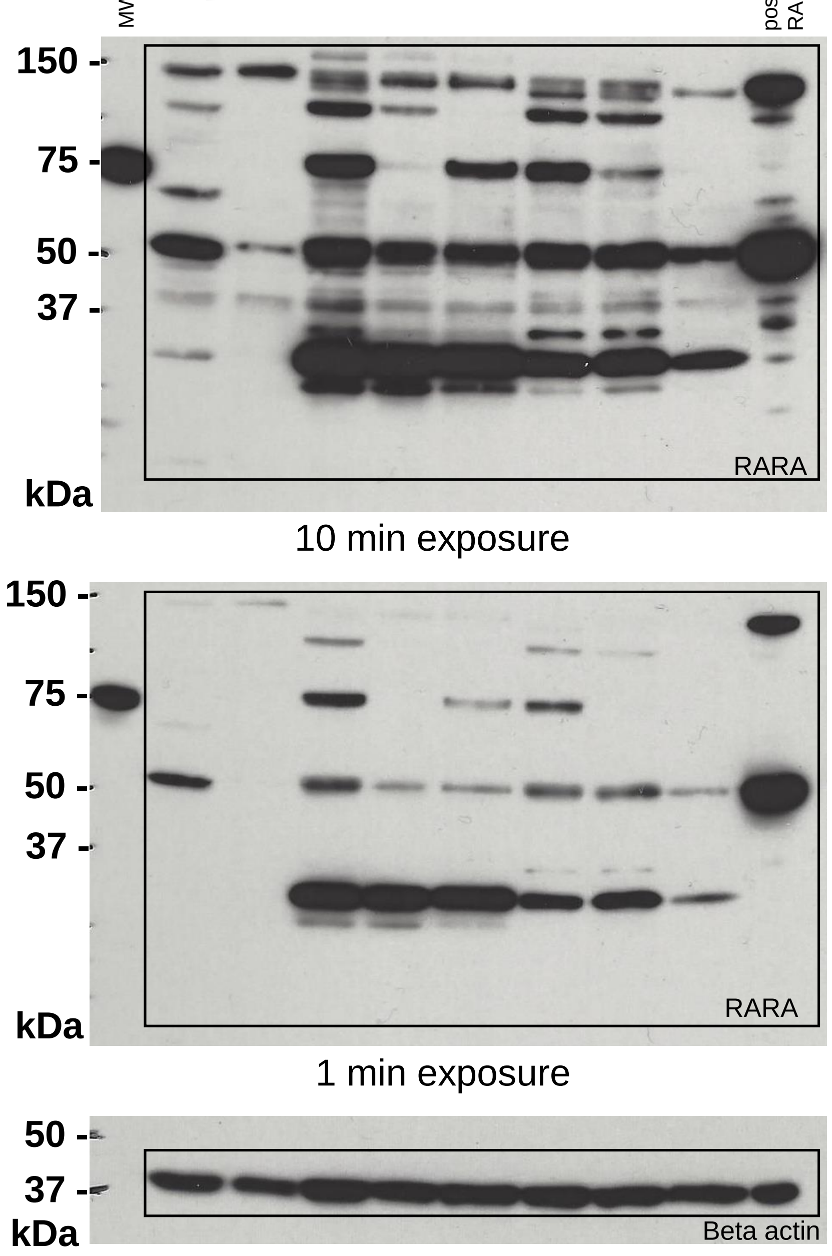


10 sec exposure

## Supplemental Figure 2

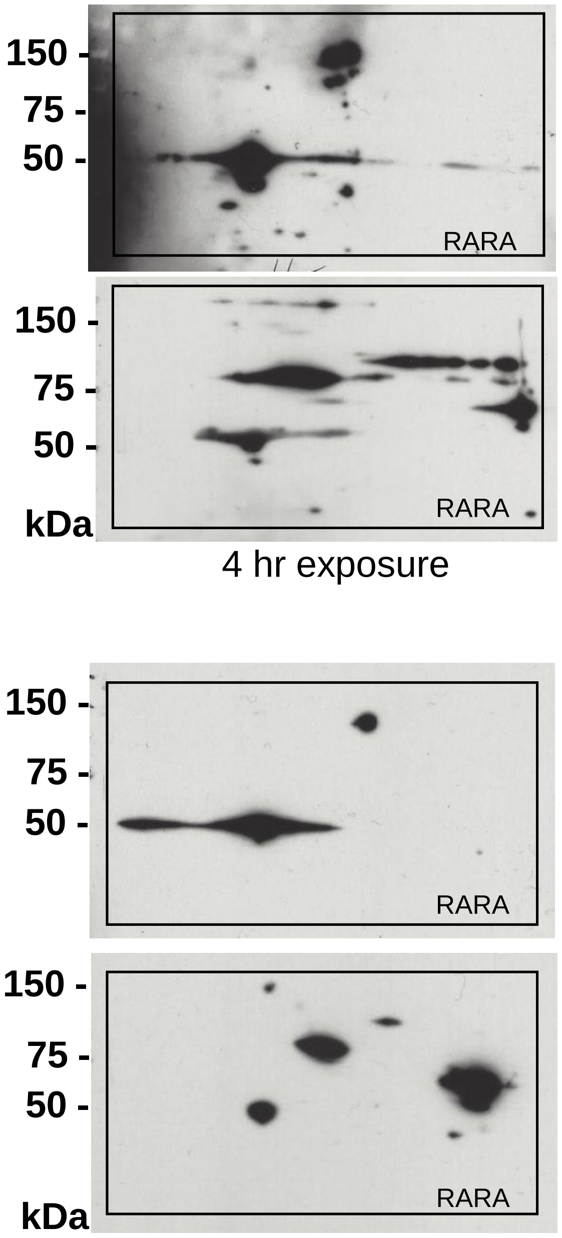


3 hr exposure

## Supplemental Figure 4

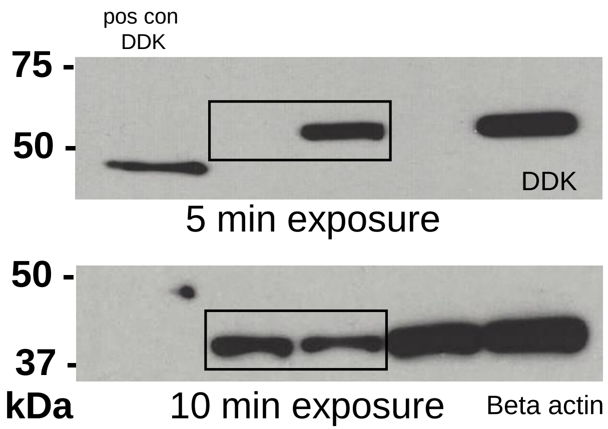


## Supplemental Figure 5 (top)

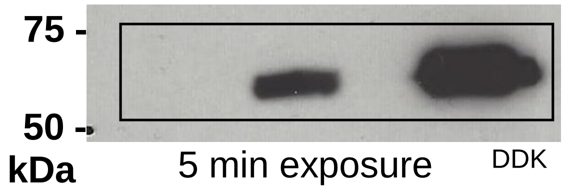


5 min exposure

## Supplemental Figure 5 (bottom)

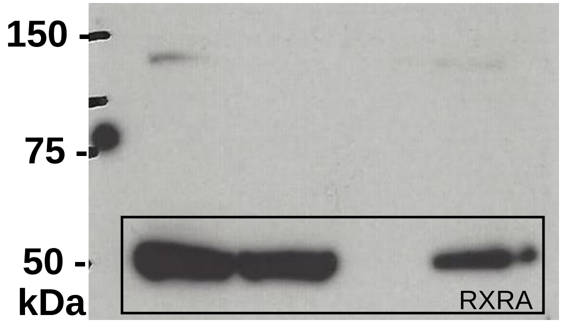


5 min exposure

## Supplemental Figure 6

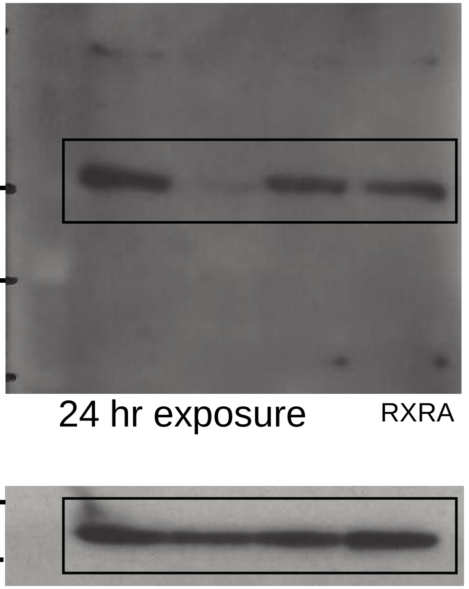


## Supplemental Figure 7

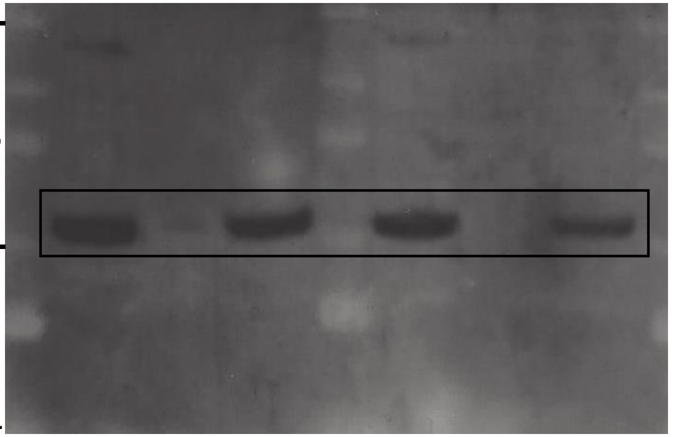


## Supplemental Figure 8

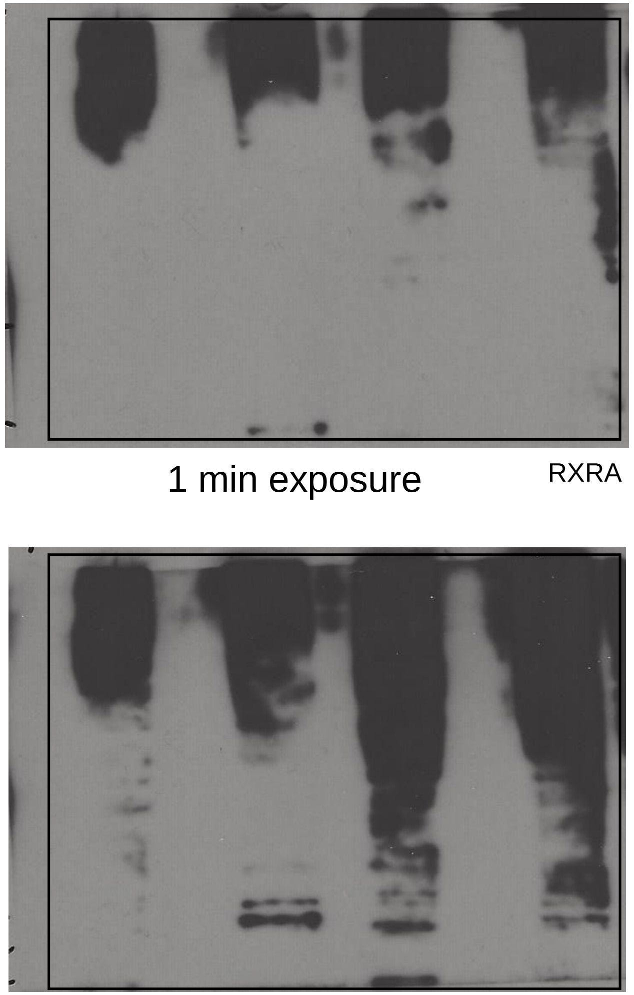

Supplement: Supplementary file 1 — Sumo1 and valosin-containing protein (VCP/p97/Cdc48) regulate retinoid receptor protein turnover– a process disrupted in glioblastoma [file 41598_2019_52696_MOESM1_ESM.pdf]
